# No optical coherence tomography changes in premanifest Huntington's disease mutation carriers far from disease onset

**DOI:** 10.1002/brb3.2592

**Published:** 2022-05-05

**Authors:** Rahel Dominique Schmid, Jana Remlinger, Mathias Abegg, Robert Hoepner, Rainer Hoffmann, Carsten Lukas, Carsten Saft, Anke Salmen

**Affiliations:** ^1^ Department of Ophthalmology Inselspital, Bern University Hospital and University of Bern Bern Switzerland; ^2^ Department of Neurology Inselspital, Bern University Hospital and University of Bern Bern Switzerland; ^3^ Graduate School for Cellular and Biomedical Sciences University of Bern Bern Switzerland; ^4^ Department of Neurology St. Josef‐Hospital Ruhr‐University Bochum Huntington Center NRW Bochum Germany; ^5^ Department of Neuropsychiatry Huntington Center South kbo‐Isar‐Amper‐Klinikum, Taufkirchen (Vils) Germany; ^6^ Institute of Neuroradiology St. Josef‐Hospital Ruhr‐University Bochum Bochum Germany

**Keywords:** biomarker, Huntington's disease, neurodegeneration, optical coherence tomography, retinal changes

## Abstract

**Background:**

Spectral‐domain optical coherence tomography (OCT) may detect retinal changes as a biomarker in neurodegenerative diseases like manifest Huntington's disease (HD). We investigate macular retinal layer thicknesses in a premanifest HD (pre‐HD) cohort and healthy controls (HC).

**Methods:**

Pre‐HD mutation carriers underwent standardized ratings and a preset macular OCT scan. Thickness values were determined for each sector of all macular retinal layers, the mean of all sectors and the mean of the inner ring (IR, 3 mm) after segmentation (Heyex segmentation batch). HC were retrospectively included from an existing database. The IR thickness of the ganglion cell layer (GCL), retinal nerve fiber layer (RNFL), GCL + inner plexiform layer (GCIPL), and total retina were included in the exploratory correlation analyses with paraclinical ratings and compared to HC.

**Results:**

The analyses comprised *n* = 24 pre‐HD participants (*n* = 10 male, *n* = 14 female) and *n *= 38 HC (*n *= 14 male, *n* = 24 female). Retinal layer parameters did not correlate with paraclinical ratings. Expected correlations between established HD biomarkers were robust. The IR thicknesses of the GCL, GCIPL, and total retina did not differ between pre‐HD and HC. The IR thickness of the RNFL was significantly higher in pre‐HD participants (pre‐HD: 23.22 μm (standard deviation 2.91), HC: 21.26 μm (1.90), *p *= .002).

**Discussion:**

In this cross‐sectional cohort of genetically determined pre‐HD participants, neurodegenerative features were not detected with retinal layer segmentation. Since our pre‐HD collective was more than 16 years before disease onset, OCT may not be sensitive enough to detect early changes.

## Graphical abstract

In this study, we investigate optical coherence tomography (OCT) data from pre‐manifest Huntington's disease mutation carriers 16 years prior to disease onset. Comparison to healthy controls and correlation with standardized clinical and paraclinical ratings did not reveal prognostic value or degenerative features. Thus, 16 years before disease onset, OCT may not be sensitive enough to detect early changes.

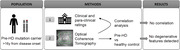

## 1 INTRODUCTION

Huntington's disease (HD) is genetically determined by a pathological expansion of cytosine‐adenin‐guanin (CAG) triplet repeats on chromosome 4 (Ghosh & Tabrizi, 2018; Paulsen et al., 2008). The consequence of this repeat expansion is an altered Huntingtin protein, which exerts neuronal dysfunction and cell death within the central nervous system (CNS; Wild et al., 2015). Affected persons experience a progressive multidimensional dysfunction involving personality, behavior, cognition, and movement (Kersten et al., 2015; Stout et al., 2011).

Curative treatment options for HD are still lacking. The promising approach of an antisense oligonucleotide (ASO) reducing mutant Huntingtin after intrathecal administration (Tabrizi, 2019) seems not to elicit clinical effects in patients in the respective Phase III trial (NCT03761849). Active treatment was thus stopped early in March 2021, the participants are currently being monitored, and the final results are pending (Roche press release, March 22, 2021). Besides ASOs, there are several other approaches potentially reducing mutant huntingtin (Wild & Tabrizi, 2017).

This underscores the need for sensitive biomarkers of neurodegeneration that can be applied in earlier stages of clinical development and can potentially detect changes earlier than clinical progression in a slowly advancing neurological disorder such as HD.

Magnetic resonance imaging (MRI) parameters for brain atrophy, such as striatal volume loss, precede clinical symptoms of HD for approximately 10 to 15 years and correlate with the estimated HD onset (Paulsen et al., 2008; Rees et al., 2013). Likewise, cortical thinning progresses with the duration of the disease (Tabrizi, 2009). However, MRI parameters of neurodegeneration are subject to limitations. An MRI investigation is time‐consuming and may be difficult for an impaired patient. The imaging quality is influenced by involuntary movements, which may particularly be relevant for the comparability of longitudinal examinations.

Optical coherence tomography (OCT) is a non‐invasive, easy‐to‐use and reproducible tool to examine the retina and the different retinal layers (Grover et al., 2009). As the retina is a neuronal structure harboring both unmyelinated axons (retinal nerve fiber layer [RNFL]) and neurons (retinal ganglion cells), OCT has already been used in several neuroinflammatory and neurodegenerative disorders (Balk et al., 2016; Bayhan et al., 2015; Behbehani et al., 2018; Coppola et al., 2015; Hart et al., 2016). In manifest HD, thinning of the temporal sector of the peripapillary RNFL (pRNFL) has been demonstrated to correlate with a longer disease duration (Kersten et al., 2015). Thinning of the temporal pRNFL occurs in various neurological disorders of different etiologies and thus does not seem to represent a specific finding (Kersten et al., 2015). For different single macular retinal layers, a thickness reduction in manifest HD has already been demonstrated. Thus, OCT is discussed as an easy and fast‐to‐perform, non‐invasive biomarker in HD (Gatto et al., 2018; Gulmez Sevim et al., 2019).

The aim of our study is (1) the investigation of macular retinal layers by spectral‐domain OCT (SD‐OCT) for the first time in a cohort of premanifest HD (pre‐HD), (2) an explorative correlation to clinical, genetic and MRI parameters, and (3) the comparison to a cohort of healthy controls (HC).

## 2 METHODS

### 2.1 Study design

The structure and presentation of the methods of this quantitative OCT study follows the Advised Protocol for OCT Study Terminology and Elements (APOSTEL) consensus recommendations (Supplementary Table S9; Cruz‐Herranz et al., 2016).

In a prospective cross‐sectional analysis between October 2011 and June 2012, participants with genetically determined pre‐HD were examined with SD‐OCT (Spectralis, HRA+OCT, Heidelberg Engineering; one device only) in the Department of Neurology, St. Josef‐Hospital, Ruhr‐University Bochum, by trained personnel (three different persons in total) in a room with dimmed artificial light. No pupil dilatation was performed.

Data of the control group were collected between September 2016 and July 2017 with SD‐OCT (Spectralis, HRA+OCT, Heidelberg Engineering, Germany) in the Department of Ophthalmology, Inselspital, by trained personnel and retrospectively used for this study.

### 2.2 Participants

#### 2.2.1 Pre‐HD cohort

Male and female adult participants without clinical HD manifestation, as defined by a diagnostic confidence level below 4, were included (Vaccarino et al., 2011). Standardized staging included quantified testing using the Unified Huntington's Disease Rating Scale (UHDRS; Huntington Study Group, 1996) motor and cognitive score, performed by trained and certified neurological personnel. The disease burden score (DBS; also called the CAG‐Index) and estimated time to disease onset in years (years to onset [YTO]) using the Langbehn formula were calculated using published algorithms (Langbehn et al., 2004; Penney et al., 1997).

Exclusion criteria were a history of ophthalmological diseases (severe myopia, papilla drusen, cataract, glaucoma, vitreous opacities, history of toxoplasmosis, retinitis, or chorioretinitis), other neurological CNS diseases, chronic diseases with potential effects on the retina (e.g., diabetes mellitus, Human Immunodeficiency Virus (HIV) infection, immunodeficiency syndromes, rheumatologic and oncological diseases, traumatic brain injury), premature birth, intake of steroids up to 8 weeks prior to examination, and claustrophobia (MRI).

#### 2.2.2 HC cohort

From the existing ophthalmological database, we retrospectively included all available participants (Table 1).

**TABLE 1:** Demographic and clinical data of the cohorts

	HC (*n* = 38)	Pre‐HD (*n* = 24)	*p*‐value
Sex (*n*)			.79
‐ male	14	10	
‐ female	24	14	
Age [years, mean (SD)]	35.75 (12.22)	39.66 (9.48)	**.04**
UHDRS motor score	n.a.	3.33 (3.91)	n.a.
UHDRS cognitive score	n.a.	337.42 (52.69)	n.a.
Caudate volume (ml)	n.a.	2.94 (0.62)	n.a.
DBS	n.a.	248.31 (75.41)	n.a.
YTO	n.a.	16.48 (7.99)	n.a

Abbreviations: DBS, disease burden score; HC, healthy controls; *n*, number; n.a., not available; pre‐HD, premanifest Huntington's disease; SD, standard deviation; UHDRS, Unified Huntington's Disease Rating Scale; YTO, years to onset.

#### 2.2.3 OCT

SD‐OCT in pre‐HD was performed by trained technical assistants of the Department of Neurology, Bochum, with Spectralis (HRA+OCT, Heidelberg Engineering). The preset high‐speed macula vertical line scan with eye‐tracking measured the thickness (in micrometre) and volume of the macula region (in cubic millimeter). Recordings were made with 61 B‐scans. The distance between the slices was 124 μm. The recorded diameter around the centered fovea was 6 mm. In this area, three circles of 1, 3, and 6‐mm diameter were set. This results in eight sectors plus the centered fovea (C0): inner superior: S1, outer superior: S2, inner temporal: T1, outer temporal: T2, inner inferior: I1, outer inferior: I2, inner nasal: N1, and outer nasal: N2.

SD‐OCT of HCs was performed independently of this study in the Department of Ophthalmology, Bern, under comparable conditions with the same device model (Spectralis, HRA+OCT, Heidelberg Engineering).

Retinal layer segmentation was performed with Heidelberg Eye Explorer (Heyex) Segmentation Batch in an automated fashion with manual correction (in case of minor inaccuracies) or exclusion of scans or sectors in case of insufficient quality (fundus image not correctly focused, OCT scan not fully displayed, segmentation not fully accomplished, other artifacts). This displayed the following structures: inner limiting membrane (ILM), RNFL, ganglion cell layer (GCL), inner plexiform layer (IPL), inner nuclear layer (INL), outer plexiform layer (OPL), outer nuclear layer (ONL), outer limiting membrane, neural layer, retinal pigment epithelium and basal membrane.

All anonymized data were exported to an Excel file for further analysis.

#### 2.2.4 MRI

MRI imaging was performed with a standardized protocol on a 1.5 Tesla scanner using sagittal high‐resolution T1‐weighted 3D MRI data (magnetization prepared rapid gradient echo) for voxel‐based morphometry as described earlier (Aylward et al., 2000, 2011; Bellenberg et al., 2015; Skodda et al., 2016). A trained neuroradiologist (CL) evaluated caudate nucleus volumes. The results were provided as a sum of caudate volumes (left + right).

### 2.3 Analysis and statistics

To describe categorical variables, we used absolute and relative frequencies. For continuous variables, we used the mean and standard deviation (SD).

For participants with left and right eye scans of sufficient quality, the means of both eyes were used for analyses. In individuals with only one available value due to quality selection, the single value was included in the analysis.

For correlation analyses and comparison to HC, the sum of the inner ring (IR) sectors (IR; S1 + T1 + I1 + N1) of the following layers was used: GCL (primary outcome), RNFL, GCL + IPL layer (GCIPL), and total retina (secondary outcomes). The exploratory unadjusted correlation analysis was calculated for these layers and age, UHDRS motor and cognitive score, caudate volume, DBS, and YTO using Pearson's correlation coefficient. Pre‐HD and HC were compared using the Mann–Whitney *U*‐test with Bonferroni correction for multiple testing of different OCT parameters or Fisher's exact test (for sex distribution).

All analyses were performed using IBM SPSS statistics, version 25 (IBM Corp., 2017).

### 2.4 Protocol approval and ethics

Written informed consent was obtained from all participants before enrollment. The examination of pre‐HD participants was conducted at the Department of Neurology, St. Josef‐Hospital, Ruhr‐University Bochum, Germany (ethics approval: ethics committee of the medical faculty of the Ruhr‐University Bochum, registration number 4090‐11) and complies with the tenets of the Declaration of Helsinki. The further use of the data in an anonymized fashion was deemed appropriate by the ethics committee after change of the principal investigator to the University of Bern. The HC group was selected from the existing database of the Department of Ophthalmology, Inselspital, University Hospital and the University of Bern, Switzerland (ethics approval: cantonal ethics committee Bern, registration number KEK‐BE 110/2015). All data were anonymized prior to analysis.

## 3 RESULTS

### 3.1 Characteristics of the pre‐HD and HC cohort

The characteristics of the pre‐HD (*n* = 24) and HC cohorts (*n* = 38) are given in Table 1. Following the Langbehn formula, on average, the participants had a calculated onset of disease of 16.47 years (SD 7.9) from the time point of investigation with low motor and high cognitive scores indicative of the pre‐HD stage. The age range of the HC cohort was broader, with pre‐HD being significantly older (*p* = .04).

### 3.2 OCT results

The mean IR thickness of the retinal layers of interest (GCL, RNFL, GCIPL, total retina) for pre‐HD and HC are summarized in Table 2. Macular sector thicknesses, the mean thickness of all sectors (6‐mm grid including the fovea) and the mean IR thickness of the pre‐HD and HC cohorts can be found in the supplement (Table S1: RNFL, Table S2: GCL, Table S3: IPL, Table S4: INL, Table S5: OPL, Table S6: ONL, Table S7: ILM‐BM (total retina), and Table S8: GCIPL).

**TABLE 2:** Mean inner ring (IR) thickness (μm) of the retinal layers of interest of healthy controls (HC) and premanifest Huntington's disease (pre‐HD) cohort

Layer	HC, mean (SD)	pre‐HD, mean (SD)	*p*‐value
GCL	52.65 (5.01)	53.99 (3.37)	.18
RNFL	21.26 (1.91)	23.22 (2.91)	**.002**
GCIPL	94.88 (8.07)	97.81 (5.48)	.10
Total retina	344.55 (16.70)	349.41 (12.54)	.16

*Note*: Mann–Whitney *U*‐test, *p* < .0125 (Bonferroni correction), statistical significance. Exact p values are given for *p* < .05.

Abbreviations: GCIPL, the sum of ganglion cell and inner plexiform layer thickness; GCL, ganglion cell layer; RNFL, retinal nerve fiber layer; SD, standard deviation.

### 3.3 Explorative correlation analyses in the pre‐HD cohort

The mean IR thicknesses of the retinal layers of interest did not show a significant correlation with age, clinical measures (UHDRS motor and cognitive score), caudate volume, DBS or YTO (Table 3A). Caudate volume positively correlated with YTO and UHDRS cognitive scores and inversely correlated with age, DBS and UHDRS motor scores (Table 3B). Even in pre‐HD, DBS and YTO correlated with the UHDRS measures (Table 3B).

**TABLE 3A:** Exploratory correlation analyses of optical coherence tomography measures and clinical, Magnetic resonance imaging (MRI) and genetic parameters

Retinal layers, mean IR thickness (μm)	Age (years)	UHDRS motor score	UHDRS cognitive score	Caudate volume (ml)	DBS	YTO
GCL	0.10 (0.65)	−0.14 (0.53)	−0.09 (0.68)	0.05 (0.81)	−0.02 (0.91)	0.04 (0.85)
RNFL	0.36 (0.09)	0.20 (0.35)	−0.03 (0.88)	0.05 (0.83)	−0.12 (0.58)	0.02 (0.93)
GCIPL	0.08 (0.71)	−0.06 (0.79)	−0.20 (0.34)	0.01 (0.98)	0.05 (0.83)	−0.01 (0.95)
Total retina	0.13 (0.53)	−0.05 (0.81)	−0.15 (0.49)	−0.17 (0.43)	0.02 (0.92)	−0.06 (0.78)

*Note*: Pearson's correlation coefficient R (p‐value), p < .05, statistical significance. Exact p‐values are given for p < .05.

Abbreviations: DBS, disease burden score; GCIPL, the sum of ganglion cell and inner plexiform layer thickness; GCL, ganglion cell layer; RNFL, retinal nerve fiber layer; SD, standard deviation; UHDRS, Unified Huntington's Disease Rating Scale; YTO, years to onset.

**TABLE 3B:** Exploratory correlation analyses of clinical, MRI and genetic parameters

	UHDRS motor score	UHDRS cognitive score	Caudate volume (ml)	DBS	YTO
Age (years)	0.32 (0.13)	−0.30 (0.16)	**−0.56 (0.005)**	0.17 (0.42)	−0.35 (0.10)
UHDRS motor score	–	**−0.50 (0.012)**	**−0.41 (0.048)**	**0.45 (0.029)**	**−0.44 (0.033)**
UHDRS cognitive score	–	–	**0.47 (0.022)**	**−0.53 (0.008)**	**0.49 (0.015)**
Caudate volume (ml)	–	–	–	**−0.64 (0.001)**	**0.64 (0.001)**
DBS	–	–	–	–	**0.95 (0.000)**

*Note*: Pearson's correlation coefficient *R* (*p*‐value), *p* < .05, statistical significance. Exact *p*‐values are given for *p* < .05.

Abbreviations: DBS, disease burden score; GCIPL, the sum of ganglion cell and inner plexiform layer thickness; GCL, ganglion cell layer; RNFL, retinal nerve fiber layer; UHDRS, Unified Huntington's Disease Rating Scale; YTO, years to onset.

### 3.4 Comparison of pre‐HD and control participants

The mean IR thicknesses for the GCL, GCIPL, and total retina did not differ between pre‐HD and HC. A slightly thicker IR RNFL was detected in pre‐HD patients (23.22 (2.91)) as compared to HC (21.26 (1.91), *p* = .002, Table 2).

## 4 DISCUSSION

This cross‐sectional study distinctly investigated macular retinal layer thicknesses in a unique albeit small pre‐HD cohort. The exploratory correlations did not indicate associations of retinal layer thicknesses with established clinical, MRI and genetic parameters. Yet, among the clinical, MRI and genetic parameters, plausible and expected associations were detected. Despite the premanifest state, caudate volumes correlated with UHDRS motor and cognitive scores, which is in line with earlier findings of subtle changes in the prodromal phase of the disease (Paulsen et al., 2008; Stout et al., 2011; Tabrizi, 2009). In summary, OCT results were not robustly different between pre‐HD and HC. The marginal but significantly higher RNFL in pre‐HD is a counterintuitive result and might be attributed to a center bias or the small HC cohort with a broader age range, which are both limitations of our study.

Altogether, the investigated OCT parameters may not be sensitive enough to serve as a reliable biomarker in this very early stage of pre‐HD. So far, few studies have described changes in subjects being more than 16 years from estimated disease onset. MRI parameters for brain atrophy, such as striatal volume loss, are reported to precede clinical symptoms of HD by approximately 10 to 15 years (Paulsen et al., 2008; Rees et al., 2013). Neurocognitive changes may occur 15 to 16 years prior to disease onset (Beste et al., 2013; Paulsen et al., 2008). Speech rate and regularity, voice changes and tapping differences were described as early changes in pre‐HD. Yet, most of these parameters are not sensitive enough in longitudinal follow‐up or were not investigated longitudinally (Rusz et al., 2014; Saft et al., 2008, 2009; Skodda et al., 2016; Tabrizi, 2012, 2019).Our collective might be too early to display detectible changes; thus, OCT investigations in a cohort closer to disease onset should be considered. Caudate volume — even in this premanifest cohort — did demonstrate associations with both clinical and genetic parameters and could be more useful in this context. This has been demonstrated earlier, and longitudinal studies for different MRI parameters were able to show progression over time correlating with clinical findings (Aylward et al., 2000, 2011; Rees et al., 2013; Tabrizi, 2012)

The cross‐sectional design of our study leaves questions on sensitivity to change for both OCT and MRI parameters. In manifest HD, different OCT parameters have already been demonstrated to be different from HC and to correlate with measures of disease progression (Andrade et al., 2016; Gulmez Sevim et al., 2019; Kersten et al., 2015) All of these studies had a cross‐sectional design. A longitudinal investigation thus still represents an unmet need. In particular, whether OCT might be used as a progression marker of neurodegeneration in treatment trials is of interest.

The HC cohort is not a matched control cohort and was retrospectively identified from an existing database in a different center, which represents another limitation of our study. Although examined with the same device and segmented with the same software by the same investigator at the same time point, we cannot exclude a potential bias herein. Yet, the inclusion of an HC cohort was deemed useful in this context as a comparator. We consider the reporting of our negative findings, although in need of further confirmation, an important contribution to the biomarker challenges in pre‐HD.

## CONFLICT OF INTEREST

RD Schmid, J Remlinger, M Abegg and R Hoffmann report no disclosures. R Hoepner received research and travel grants from Novartis and Biogen Idec. He also received speaker honoraria from Biogen, Novartis, Merck and Almirall and research support from the Swiss MS Society. C Lukas received a research grant from the German Federal Ministry for Education and Research, BMBF, German Competence Network Multiple Sclerosis (KKNMS), grant no. 01GI1601I, and received consulting and speaker's honoraria from Biogen, Bayer, Daiichi Sanykyo, Merck Serono, Novartis, Sanofi, Genzyme and TEVA. C Saft reports personal fees/honoraria from Teva Pharma GmbH, as well as nonfinancial support and other support from ENROLL‐HD study (CHDI), PRIDE‐HD (TEVA), LEGATO (TEVA), and Amaryllis (Pfizer), ASO (IONIS Pharmaceuticals, Roche AG and WAVE), RROOF‐HD (Prilenia) for the conducting of studies and grants from Biogen all outside the submitted work and without relevance to the article. A Salmen received speaker honoraria and/or travel compensation for activities with Bristol Myers Squibb, CSL Behring, Novartis and Roche and research support from the Baasch Medicus Foundation and the Swiss MS Society, all not related to this work.

### PEER REVIEW

The peer review history for this article is available at https://publons.com/publon/10.1002/brb3.2592

## Supporting information

Supporting information.

## Data Availability

The datasets supporting the conclusions of this article are available to any qualified researcher upon reasonable request from the corresponding author.

## References

[brb32592-bib-0001] Andrade, C. , Beato, J. , Monteiro, A. , Costa, A. , Penas, S. , Guimarães, J. , Reis, F. F. , & Garrett, C. (2016). Spectral‐domain optical coherence tomography as a potential biomarker in Huntington's disease. Movement Disorders, 31(3), 377–383. 10.1002/mds.26486 26853218

[brb32592-bib-0002] Aylward, E. H. , Codori, A. M. , Rosenblatt, A. , Sherr, M. , Brandt, J. , Stine, O. C. , Barta, P. E. , Pearlson, G. D. , & Ross, C. A. (2000). Rate of caudate atrophy in presymptomatic and symptomatic stages of Huntington's disease. Movement Disorders, 15(3), 552–560. 10.1002/1531-8257(200005)15:3<552::AID-MDS1020>3.0.CO;2-P 10830423

[brb32592-bib-0003] Aylward, E. H. , Nopoulos, P. C. , Ross, C. A. , Langbehn, D. R. , Pierson, R. K. , Mills, J. A. , Johnson, H. J. , Magnotta, V. A. , Juhl, A. R. , & Paulsen, J. S. (2011). Longitudinal change in regional brain volumes in prodromal Huntington disease. Journal of Neurology, Neurosurgery, and Psychiatry, 82(4), 405–410. 10.1136/jnnp.2010.208264 20884680 PMC3105627

[brb32592-bib-0004] Balk, L. J. , Cruz‐Herranz, A. , Albrecht, P. , Arnow, S. , Gelfand, J. M. , Tewarie, P. , Killestein, J. , Uitdehaag, B. M. J. , Petzold, A. , & Green, A. J. (2016). Timing of retinal neuronal and axonal loss in MS: A longitudinal OCT study. Journal of Neurology, 263(7), 1323–1331. 10.1007/s00415-016-8127-y 27142714 PMC4929170

[brb32592-bib-0005] Bayhan, H. A. , Aslan Bayhan, S. , Celikbilek, A. , Tanik, N. , & Gürdal, C. (2015). Evaluation of the chorioretinal thickness changes in Alzheimer's disease using spectral‐domain optical coherence tomography. Clinical and Experimental Ophthalmology, 43(2), 145–151. 10.1111/ceo.12386 24995484

[brb32592-bib-0006] Behbehani, R. , Adnan, H. , Al‐Hassan, A. A. , Al‐Salahat, A. , & Alroughani, R. (2018). Predictors of retinal atrophy in multiple sclerosis: A longitudinal study using spectral domain optical coherence tomography with segmentation analysis. Multiple Sclerosis and Related Disorders, 21, 56–62. 10.1016/j.msard.2018.02.010 29459346

[brb32592-bib-0007] Bellenberg, B. , Schneider, R. , Weiler, F. , Suchan, B. , Haghikia, A. , Hoffjan, S. , Gold, R. , Köster, O. , & Lukas, C. (2015). Cervical cord area is associated with infratentorial grey and white matter volume predominantly in relapsing‐remitting multiple sclerosis: A study using semi‐automated cord volumetry and voxel‐based morphometry. Multiple Sclerosis and Related Disorders, 4(3), 264–272. 10.1016/j.msard.2015.04.003 26008944

[brb32592-bib-0008] Beste, C. , Stock, A. ‐K. , Ness, V. , Hoffmann, R. , Lukas, C. , & Saft, C. (2013). A novel cognitive‐neurophysiological state biomarker in premanifest Huntington's disease validated on longitudinal data. Scientific Reports, 3(1), 1797–1797. 10.1038/srep01797 23652721 PMC3647202

[brb32592-bib-0009] Coppola, G. , Di Renzo, A. , Ziccardi, L. , Martelli, F. , Fadda, A. , Manni, G. , Barboni, P. , Pierelli, F. , Sadun, A. A. , & Parisi, V. (2015). Optical coherence tomography in Alzheimer's disease: A meta‐analysis. PLoS One, 10(8), e0134750–e0134750. 10.1371/journal.pone.0134750 26252902 PMC4529274

[brb32592-bib-0010] Cruz‐Herranz, A. , Balk, L. J. , Oberwahrenbrock, T. , Saidha, S. , Martinez‐Lapiscina, E. H. , Lagreze, W. A. , Schuman, J. S. , Villoslada, P. , Calabresi, P. , Balcer, L. , Petzold, A. , Green, A. J. , Paul, F. , Brandt, A. U. , & Albrecht, P. (2016). The APOSTEL recommendations for reporting quantitative optical coherence tomography studies. Neurology, 86(24), 2303–2309. 10.1212/WNL.0000000000002774 27225223 PMC4909557

[brb32592-bib-0011] Gatto, E. , Parisi, V. , Persi, G. , Rey, E. F. , Cesarini, M. , Etcheverry, J. L. , Rivera, P. , & Squitieri, F. (2018). Optical coherence tomography (OCT) study in Argentinean Huntington's disease patients. The International Journal of Neuroscience, 128(12), 1157–1162. 10.1080/00207454.2018.1489807 29912591

[brb32592-bib-0012] Ghosh, R. , & Tabrizi, S. J. (2018). Clinical features of Huntington's disease. Advances in Experimental Medicine and Biology, 1049, 1–28. 10.1007/978-3-319-71779-1_1 29427096

[brb32592-bib-0013] Grover, S. , Murthy, R. K. , Brar, V. S. , & Chalam, K. V. (2009). Normative data for macular thickness by high‐definition spectral‐domain optical coherence tomography (spectralis). American Journal of Ophthalmology, 148(2), 266–271. 10.1016/j.ajo.2009.03.006 19427616

[brb32592-bib-0014] Gulmez Sevim, D. , Unlu, M. , Gultekin, M. , & Karaca, C. (2019). Retinal single‐layer analysis with optical coherence tomography shows inner retinal layer thinning in Huntington's disease as a potential biomarker. International Ophthalmology, 39(3), 611–621. 10.1007/s10792-018-0857-7 29435796

[brb32592-bib-0015] Hart, N. J. , Koronyo, Y. , Black, K. L. , & Koronyo‐Hamaoui, M. (2016). Ocular indicators of Alzheimer's: Exploring disease in the retina. Acta Neuropathologica, 132(6), 767–787. 27645291 10.1007/s00401-016-1613-6 PMC5106496

[brb32592-bib-0016] Huntington Study Group . (1996). Unified Huntington's disease rating scale: Reliability and consistency. Movement Disorders, 11(2), 136–142. 10.1002/mds.870110204 8684382

[brb32592-bib-0017] Kersten, H. M. , Danesh‐Meyer, H. V. , Kilfoyle, D. H. , & Roxburgh, R. H. (2015). Optical coherence tomography findings in Huntington's disease: A potential biomarker of disease progression. Journal of Neurology, 262(11), 2457–2465. 10.1007/s00415-015-7869-2 26233693

[brb32592-bib-0018] Langbehn, D. , Brinkman, R. , Falush, D. , Paulsen, J. , Hayden, M. R. , & International Huntington's Disease Collaborative Group . (2004). A new model for prediction of the age of onset and penetrance for Huntington's disease based on CAG length. Clinical Genetics, 65, 267–277. 15025718 10.1111/j.1399-0004.2004.00241.x

[brb32592-bib-0019] Paulsen, J. , Langbehn, D. , Stout, J. , Aylward, E. , Ross, C. A. , Nance, M. , Guttman, M. , Johnson, S. , MacDonald, M. , Beglinger, L. J. , Duff, K. , Kayson, E. , Biglan, K. , Shoulson, I. , Oakes, D. , & Hayden, M. (2008). Detection of Huntington's disease decades before diagnosis: The PREDICT‐HD study. Journal of Neurology, Neurosurgery, and Psychiatry, 79, 874–880. 18096682 10.1136/jnnp.2007.128728 PMC2569211

[brb32592-bib-0020] Penney, J. B. , Vonsattel, J. ‐P. , Macdonald, M. E. , Gusella, J. F. , & Myers, R. H. (1997). CAG repeat number governs the development rate of pathology in Huntington's disease. Annals of Neurology, 41(5), 689–692. 10.1002/ana.410410521 9153534

[brb32592-bib-0021] Rees, E. M. , Scahill, R. I. , & Hobbs, N. Z. (2013). Longitudinal neuroimaging biomarkers in Huntington's disease. Journal of Huntington's Disease, 2(1), 21–39. 10.3233/JHD-120030 25063427

[brb32592-bib-0022] Rusz, J. , Saft, C. , Schlegel, U. , Hoffman, R. , & Skodda, S. (2014). Phonatory dysfunction as a preclinical symptom of Huntington disease. PLoS One, 9(11), e113412–e113412. 25409322 10.1371/journal.pone.0113412 PMC4237453

[brb32592-bib-0023] Saft, C. , Andrich, J. , Meisel, N. ‐M. , Przuntek, H. , & Müller, T. (2006). Assessment of simple movements reflects impairment in Huntington's disease. Movement Disorders: Official Journal of the Movement Disorder Society, 21(8), 1208–1212. 10.1002/mds.20939 16700032

[brb32592-bib-0024] Saft, C. , Kosinski, C. M. , & Landwehrmeyer, G. B. (2009). Fortschritte in Früh‐ und Verlaufsdiagnostik bei Morbus Huntington. Aktuelle Neurologie, 36(10), 506–523.

[brb32592-bib-0025] Skodda, S. , Grönheit, W. , Lukas, C. , Bellenberg, B. , Von Hein, S. M. , Hoffmann, R. , & Saft, C. (2016). Two different phenomena in basic motor speech performance in premanifest Huntington disease. Neurology, 86(14), 1329–1335. 10.1212/WNL.0000000000002550 26962067

[brb32592-bib-0026] Stout, J. C. , Paulsen, J. S. , Queller, S. , Solomon, A. C. , Whitlock, K. B. , Campbell, J. C. , Carlozzi, N. , Duff, K. , Beglinger, L. J. , Langbehn, D. R. , Johnson, S. A. , Biglan, K. M. , Aylward, E. H. , & The PREDICT‐HD Investigators and Coordinators of the Huntington Study Group . (2011). Neurocognitive signs in prodromal Huntington disease. Neuropsychology, 25(1), 1–14. 10.1037/a0020937 20919768 PMC3017660

[brb32592-bib-0027] Tabrizi, S. J. , Langbehn, D. R. , Leavitt, B. R. , Roos, R. A. C. , Durr, A. , Craufurd, D. , Kennard, C. , Hicks, S. L. , Fox, N. C. , Scahill, R. I. , Borowsky, B. , Tobin, A. J. , Rosas, H. D. , Johnson, H. , Reilmann, R. , Landwehrmeyer, B. , & Stout, J. C. (2009). Biological and clinical manifestations of Huntington's disease in the longitudinal TRACK‐HD study: Cross‐sectional analysis of baseline data. The Lancet Neurology, 8(9), 791–801. 10.1016/S1474-4422(09)70170-X 19646924 PMC3725974

[brb32592-bib-0028] Tabrizi, S. J. , Leavitt, B. R. , Landwehrmeyer, G. B. , Wild, E. J. , Saft, C. , Barker, R. A. , Blair, N. F. , Craufurd, D. , Priller, J. , Rickards, H. , Rosser, A. , Kordasiewicz, H. B. , Czech, C. , Swayze, E. E. , Norris, D. A. , Baumann, T. , Gerlach, I. , Schobel, S. A. , Paz, E. , … Lane, R. M. (2019). Targeting Huntingtin expression in patients with Huntington's disease. New England Journal of Medicine, 380(24), 2307–2316. 10.1056/NEJMoa1900907 31059641

[brb32592-bib-0029] Tabrizi, S. J. , Reilmann, R. , Roos, R. A. C. , Durr, A. , Leavitt, B. , Owen, G. , Jones, R. , Johnson, H. , Craufurd, D. , Hicks, S. L. , Kennard, C. , Landwehrmeyer, B. , Stout, J. C. , Borowsky, B. , Scahill, R. I. , Frost, C. , Langbehn, D. R. , & TRACK‐HD investigators . (2012). Potential endpoints for clinical trials in premanifest and early Huntington's disease in the TRACK‐HD study: Analysis of 24 month observational data. The Lancet Neurology, 11(1), 42–53. 10.1016/S1474-4422(11)70263-0 22137354

[brb32592-bib-0030] Vaccarino, A. L. , Anderson, K. , Borowsky, B. , Duff, K. , Giuliano, J. , Guttman, M. , Ho, A. K. , Orth, M. , Paulsen, J. S. , Sills, T. , Van Kammen, D. P. , & Evans, K. R. (2011). An item response analysis of the motor and behavioral subscales of the unified Huntington's disease rating scale in Huntington disease gene expansion carriers. Movement Disorders, 26(5), 877–884. 10.1002/mds.23574 21370269 PMC3157755

[brb32592-bib-0031] Wild, E. J. , Boggio, R. , Langbehn, D. , Robertson, N. , Haider, S. , Miller, J. R. C. , Zetterberg, H. , Leavitt, B. R. , Kuhn, R. , Tabrizi, S. J. , Macdonald, D. , & Weiss, A. (2015). Quantification of mutant huntingtin protein in cerebrospinal fluid from Huntington's disease patients. Journal of Clinical Investigation, 125(5), 1979–1986. 10.1172/JCI80743 25844897 PMC4463213

[brb32592-bib-0032] Wild, E. J. , & Tabrizi, S. J. (2017). Therapies targeting DNA and RNA in Huntington's disease. The Lancet Neurology, 16(10), 837–847. 10.1016/S1474-4422(17)30280-6 28920889 PMC5604739

