# Supplementary material for: No optical coherence tomography changes in premanifest Huntington's disease mutation carriers far from disease onset
Source: Brain Behav. 2022 May 5;12(6):e2592. doi: 10.1002/brb3.2592 (PMC9226796; doi:10.1002/brb3.2592)
Supplement: Supplementary file 1 — Supporting information. [file BRB3-12-e2592-s001.docx]

# Supplementary material

**Table S1.** RNFL thickness (µm) of each sector of healthy controls (HC) and premanifest Huntington’s disease (pre-HD) cohort.

| **Sectors** | **HC, mean (SD)** | **Pre-HD, mean (SD)** | **p-value** |
| --- | --- | --- | --- |
| C0 | 12.34 (2.10) | 13.56 (1.83) | nt |
| N1 | 19.90 (1.72) | 23.13 (7.48) | nt |
| N2 | 49.29 (7.56) | 53.06 (8.79) | nt |
| S1 | 24.47 (2.91) | 25.48 (2.88) | nt |
| S2 | 38.35 (5.51) | 38.50 (5.10) | nt |
| T1 | 16.15 (1.07) | 17.25 (0.82) | nt |
| T2 | 17.85 (1.26) | 18.54 (1.59) | nt |
| I1 | 24.61 (3.14) | 27.04 (2.48) | nt |
| I2 | 39.92 (6.44) | 43.71 (8.49) | nt |
| all sectors | 26.97 (2.86) | 28.92 (3.77) | nt |
| inner ring | 21.26 (1.91) | 23.22 (2.91) | **0.002** |

Mann-Whitney-U test, p<0.0125 (Bonferroni correction), statistical significance. Exact p-values are given for p<0.05. Abbreviations: HC – healthy controls; nt – not tested; pre-HD – premanifest Huntington’s disease; RNFL – retinal nerve fiber layer.

**Table S2.** GCL thickness (µm) of each sector of healthy controls (HC) and premanifest Huntington’s disease (pre-HD) cohort.

| **Sectors** | **HC, mean (SD)** | **Pre-HD, mean (SD)** | **p-value** |
| --- | --- | --- | --- |
| C0 | 15.03 (3.60) | 17.15(4.83) | nt |
| N1 | 53.68 (5.57) | 55.42 (3.76) | nt |
| N2 | 40.01 (4.06) | 39.31 (4.16) | nt |
| S1 | 53.86 (4.99) | 55.00 (3.44) | nt |
| S2 | 35.71 (3.48) | 35.10 (3.18) | nt |
| T1 | 49.75 (5.49) | 51.00 (3.30) | nt |
| T2 | 38.07 (4.77) | 37.35 (3.06) | nt |
| I1 | 53.30 (4.77) | 54.56 (3.91) | nt |
| I2 | 34.42 (3.14) | 33.00 (3.03) | nt |
| all sectors | 41.54 (3.71) | 41.99 (2.91) | nt |
| inner ring | 52.65 (5.01) | 53.99 (3.37) | 0.18 |

Mann-Whitney-U test, p<0.0125 (Bonferroni correction), statistical significance. Abbreviations: GCL – ganglion cell layer; HC – healthy controls; nt – not tested; pre-HD – premanifest Huntington’s disease.

**Table S3.** IPL thickness (µm) of each sector of healthy controls (HC) and premanifest Huntington’s disease (pre-HD) cohort.

| **Sectors** | **HC, mean (SD)** | **Pre-HD, mean (SD)** |
| --- | --- | --- |
| C0 | 21.20 (3.14) | 23.98 (3.99) |
| N1 | 42.29 (3.45) | 43.71 (2.74) |
| N2 | 31.00 (3.01) | 30.79 (3.11) |
| S1 | 42.93 (3.66) | 44.35 (2.97) |
| S2 | 29.36 (3.13) | 27.54 (2.26) |
| T1 | 40.92 (2.88) | 42.54 (2.78) |
| T2 | 33.27 (2.30) | 34.04 (1.97) |
| I1 | 42.76 (4.04) | 44.67 (2.33) |
| I2 | 26.96 (4.76) | 26.00 (2.34) |
| all sectors mean | 34.52 (2.43) | 35.29 (1.95) |
| inner ring mean | 42.23 (3.27) | 43.82 (2.27) |

Abbreviations: HC – healthy controls; IPL – inner plexiform layer; pre-HD – premanifest Huntington’s disease.

**Table S4.** INL thickness (µm) of each sector of healthy controls (HC) and premanifest Huntington’s disease (pre-HD) cohort.

| **Sectors** | **HC, mean (SD)** | **Pre-HD, mean (SD)** |
| --- | --- | --- |
| C0 | 18.53 (4.72) | 19.33 (4.66) |
| N1 | 42.76 (3.71) | 41.06 (3.56) |
| N2 | 35.75 (2.56) | 33.35 (2.75) |
| S1 | 41.80 (3.37) | 41.23 (3.73) |
| S2 | 33.67 (2.57) | 33.40 (2.49) |
| T1 | 38.61 (3.33) | 37.85 (2.45) |
| T2 | 34.20 (2.76) | 32.19 (2.11) |
| I1 | 41.65 (3.89) | 38.60 (4.04) |
| I2 | 33.41 (2.51) | 32.06 (2.74) |
| all sectors | 35.59 (2.34) | 34.34 (2.05) |
| inner ring | 41.20 (2.84) | 39.69 (2.58) |

Abbreviations: HC – healthy controls; INL – inner nuclear layer pre-HD – premanifest Huntington’s disease.

**Table S5.** OPL thickness (µm) of each sector of healthy controls (HC) and premanifest Huntington’s disease (pre-HD) cohort.

| **Sectors** | **HC, mean (SD)** | **Pre-HD, mean (SD)** |
| --- | --- | --- |
| C0 | 25.06 (5.26) | 26.68 (5.31) |
| N1 | 35.01 (9.92) | 36.67 (9.50) |
| N2 | 28.61 (3.03) | 30.54 (3.32) |
| S1 | 31.58 (4.19) | 37.10 (6.84) |
| S2 | 25.75 (1.78) | 28.67 (3.17) |
| T1 | 30.45 (5.52) | 34.10 (4.98) |
| T2 | 26.17 (2.26) | 28.75 (2.57) |
| I1 | 32.29 (4.70) | 34.71 (7.09) |
| I2 | 26.34 (2.26) | 27.50 (2.84) |
| all sectors | 29.03 (2.58) | 31.64 (2.82) |
| inner ring | 32.33 (3.84) | 35.65 (3.90) |

Abbreviations: HC – healthy controls; OPL – outer plexiform layer; pre-HD – premanifest Huntington’s disease.

**Table S6.** ONL thickness (µm) of each sector of healthy controls (HC) and premanifest Huntington’s disease (pre-HD) cohort.

| **Sectors** | **HC, mean (SD)** | **Pre-HD, mean (SD)** |
| --- | --- | --- |
| C0 | 96.09 (10.28) | 91.73 (9.47) |
| N1 | 74.43 (14.52) | 72.44 (13.15) |
| N2 | 59.80 (8.95) | 57.25 (9.45) |
| S1 | 74.40 (9.36) | 67.89 (12.16) |
| S2 | 65.08 (7.84) | 81.23 (96.41) |
| T1 | 76.88 (9.20) | 73.35 (8.69) |
| T2 | 61.20 (7.18) | 58.71 (5.18) |
| I1 | 70.80 (9.65) | 68.65 (9.21) |
| I2 | 55.60 (6.61) | 53.77 (5.15) |
| all sectors | 70.48 (8.02) | 69.45 (12.13) |
| inner ring | 74.13 (8.94) | 70.58 (7.94) |

Abbreviations: HC – healthy controls; ONL – outer nuclear layer; pre-HD – premanifest Huntington’s disease.

**Table S7.** ILM-BM thickness (µm) of each sector of healthy controls (HC) and premanifest Huntington’s disease (pre-HD) cohort.

| **Sectors** | **HC, mean (SD)** | **Pre-HD, mean (SD)** | **p-value** |
| --- | --- | --- | --- |
| C0 | 273.99 (19.72) | 283.33 (21.74) | nt |
| N1 | 349.43 (17.53) | 354.88 (13.76) | nt |
| N2 | 323.24 (17.48) | 323.75 (15.19) | nt |
| S1 | 350.24 (17.37) | 353.92 (12.79) | nt |
| S2 | 306.11 (15.71) | 304.98 (12.31) | nt |
| T1 | 333.13 (15.94) | 338.65 (11.46) | nt |
| T2 | 288.42 (14.54) | 288.15 (10.68) | nt |
| I1 | 345.39 (17.45) | 350.19 (13.46) | nt |
| I2 | 294.86 (14.60) | 295.06 (13.73) | nt |
| all sectors | 318.31 (14.56) | 321.43 (11.58) | nt |
| inner ring | 344.55 (16.70) | 349.41 (12.54) | 0.16 |

Mann-Whitney-U test, p<0.0125 (Bonferroni correction), statistical significance. Abbreviations: BM – Bruch’s membrane; HC – healthy controls; ILM – inner limiting membrane; nt – not tested; pre-HD – premanifest Huntington’s disease.

**Table S8.** GCIPL thickness (µm) of each sector of healthy controls (HC) and premanifest Huntington’s disease (pre-HD) cohort.

| **Sectors** | **HC, mean (SD)** | **Pre-HD, mean (SD)** | **p-value** |
| --- | --- | --- | --- |
| C0 | 36.22 (6.38) | 41.13 (8.64) | nt |
| N1 | 95.97 (8.73) | 99.13 (6.04) | nt |
| N2 | 71.01 (6.99) | 70.10 (7.14) | nt |
| S1 | 96.79 (8.28) | 99.35 (6.21) | nt |
| S2 | 65.07 (6.07) | 62.65 (5.34) | nt |
| T1 | 90.67 (7.87) | 93.54 (5.68) | nt |
| T2 | 70.34 (9.27) | 71.40 (4.59) | nt |
| I1 | 96.07 (8.47) | 99.23 (5.92) | nt |
| I2 | 61.38 (6.61) | 59.00 (5.11) | nt |
| all sectors | 76.06 (5.97) | 77.28 (4.78) | nt |
| inner ring | 94.88 (8.07) | 97.81 (5.48) | 0.10 |

Mann-Whitney-U test, p<0.0125 (Bonferroni correction), statistical significance. Abbreviations: GCIPL – sum of ganglion cell and inner plexiform layer thickness; HC – healthy controls; nt – not tested; pre-HD – premanifest Huntington’s disease.

**Table S9.** Nine-point Advised Protocol for OCT Study Terminology and Elements checklist (APOSTEL)^18^

| **Item** | **Recommendation** | **Described on page** |
| --- | --- | --- |
| Study protocol | OCT operating sites and graders | 6 |
|  | Timing of OCT compared to other measurements | 8 |
|  | Inclusion and exclusion criteria | 6 |
| Acquisition device | For all OCT devices used, report data on: |  |
|  | Manufacturer | 7 |
|  | Model | 7 |
|  | Version | 7 |
|  | Software version | 7 |
| Acquisition Settings | Clearly describe the settings in which OCT scans were obtained: |  |
|  | Room light conditions | 6 |
|  | Pupils dilated before examination (y/n) | 6 |
|  | Number of operators and devices | 6 |
| Scanning protocol | Type of scan (circular, volume, star, line, other) | 7 |
|  | Location | 7 |
|  | Scan parameters (with or without eye tracking) | 7 |
| Funduscopic imaging | - | - |
| Postacquisition data selection | Describe image selection process, including: |  |
|  | Quality control criteria (i.e., OSCAR-IB17 or other criteria) | 8 |
|  | Postacquisition discard (number and criteria) | 11 |
|  | Eye selection strategy (if applicable) | 8 |
| Postacquisition analysis | Describe all postacquisition steps: |  |
|  | Software used for processing scans and segmentation | 7, 8 |
|  | Which individual retinal layers were segmented/included | 7 |
|  | Method of segmentation | 7 |
|  | How potential bias was addressed in the case of manual segmentation | - |
|  | Grid used for data extraction (size, shape, selected sections) | 7 |
| Nomenclature and abbreviations | Anatomical structures analyzed | 7 |
|  | Units of provided measurements (e.g., volume or thickness) | 7 |
| Statistical approach | Statistical models used for the analyses of OCT data | 7 |
|  | Whether data were analyzed by eye or by patient | 10 |
